# Tailoring Ovarian Cancer Treatment: Implications of *BRCA1/2* Mutations

**DOI:** 10.3390/cancers11030416

**Published:** 2019-03-23

**Authors:** Ainhoa Madariaga, Stephanie Lheureux, Amit M. Oza

**Affiliations:** Division of Medical Oncology & Hematology, Princess Margaret Cancer Center, Toronto, ON M5G 2M9, Canada; ainhoa.madariaga@uhn.ca (A.M.); stephanie.lheureux@uhn.ca (S.L.)

**Keywords:** ovarian cancer, *BRCA* mutation, homologous recombination, treatment, platinum, PARP inhibitor, resistance

## Abstract

High grade serous ovarian cancer (HGSOC) is the most common epithelial ovarian cancer, harbouring more than 20% germline or somatic mutations in the tumour suppressor genes *BRCA1* and *BRCA2*. These genes are involved in both DNA damage repair process via homologous recombination (HR) and transcriptional regulation. *BRCA* mutation confers distinct characteristics, including an increased response to DNA-damaging agents, such us platinum chemotherapy and poly-ADP ribose polymerase inhibitors (PARPi). However, several mechanisms of resistance to these agents have been described, including increased HR capacity through reverse *BRCA* mutations, non-homologous end-joint (NHEJ) repair alterations and drug efflux pumps. Current treatments of ovarian cancer including surgery, chemotherapy, targeted treatment and maintenance strategies, as well as resistance mechanisms will be reviewed, focusing on future trends with respect to *BRCA* mutation carriers.

## 1. Introduction

Contemporary cancer statistics highlight that ovarian cancer (OC) remains the most lethal gynecological malignancy in developed countries [1]; it is the seventh most common cancer in women worldwide, accounting for nearly 4% of all new cases of female cancers [2]. Approximately 90% of all OC cases are epithelial and fall within five distinct subtypes—high and low grade serous, endometrioid, clear cell and mucinous carcinomas [3,4]. Of these, high grade serous ovarian cancer (HGSOC) is the most common subtype, often diagnosed in Stage III (51%) and IV (29%), when disease has spread beyond the peritoneum leading to a modest 5-year-cause specific survival of 42% and 26%, respectively [5].

Standard front-line treatment for advanced OC has remained cytoreductive surgery with the goal of no residual disease (R0), followed by the combination of platinum and taxane chemotherapy. Response to front-line therapy helps define predictive response to second-line and subsequent therapies as OC typically follows a response-relapse pattern behavior. Therefore, at the time of recurrence, the platinum free interval (PFI)—time between the last dose of platinum based therapy and recurrence—is used to guide treatment decision [6,7]. OC is classified as *platinum resistant* if relapse occurs within six months and considered *platinum sensitive* if relapse occurs beyond six months [6,7,8]. This definition has been updated following the Fifth Ovarian Cancer Consensus Conference to therapy-free interval (TFI) [9]. TFI now includes delineation of type of therapeutic intervention in subsequent lines of therapy such as TFIp (PFI—platinum free interval), TFInp (non-PFI) and TFIb (biologic agent-free interval) and incorporates consideration of histology, *BRCA1/2* mutation status (*BRCA*m), number/type of previous therapies, outcome of prior surgery and reported symptoms [9].

Incorporating these elements is critical as it is well appreciated that whilst some genomic characteristics may be shared across subtypes, disease biology and preponderance of mutations/alterations are distinct between OC subtypes. HGSOC are characterized by ubiquitous *TP53* mutations, and significant focal DNA copy number alterations [10], while low grade serous OC is defined as *TP53* wild-type [11]. Approximately 15–20% of HGSOCs may be inherited with the most common germline mutations related to alterations in breast cancer 1 (*BRCA1*) and breast cancer 2 (*BRCA2*) genes [10]. The frequency and type of mutations differs among populations [12]; with the highest frequency being reported in Ashkenazi Jewish population, up to 2% of germline founder *BRCA1/2* mutations [13]. *BRCA1* mutations are more common than *BRCA2* in OC, although the ratio of *BRCA*1 to *BRCA*2 varies between populations [12]. A cohort study with 445 women did not find an association between ethnicity, likelihood of having breast or ovarian cancers and overall survival (OS) [14]. In addition, somatic *BRCA1/2*m can be identified in ~3% HGSOC, not related to germline mutation [10]. More broadly, defects in homologous recombination (HR), which is involved in repair of DNA damage and DNA replication, are common in HGSOC and detected in ~50% of the patients [10,15]. This review will focus on HGSOC treatment and potential differences associated with *BRCA*m status.

### 1.1. BRCA1/2 Mutations, HR Genes and High Grade Serous Ovarian Cancer

*BRCA1* and *BRCA2* genes are located on chromosomes 13 and 17, respectively. They are classified as tumour suppressors and are implicated in double strand DNA (dsDNA) break repair via HR to maintain genomic integrity. Additionally, *BRCA1/2* are involved in transcriptional co-regulation, chromatin remodeling and cell cycle control. Cells lacking BRCA accumulate chromosomal abnormalities, resulting in chromosomal instability [16,17]. Germline *BRCA1/2* mutations (g*BRCA*m) confer an increased risk of breast and HGSOC, and, to a lesser extent, of other type of cancers such as certain pancreatic [18], prostate [19], stomach and colon cancers [20]. A meta-analysis involving 22 studies with >8000 cases showed that the average cumulative risk for OC by the age of 70 was 39% and 11% in *BRCA1*m and *BRCA2*m carriers, respectively [21]. However, the majority of HGSOC are sporadic, and a proportion of these will have alteration in BRCA function through somatic mutations in *BRCA1/2* genes or as a result of methylation [10].

In addition to *BRCA1/2*, other germline HR related mutations have been identified in up to ~6% of HGSOC from a case series [22]. The Fanconi Anemia pathway controls DNA repair via HR, and several genes in this pathway, such as *BRIP1, RAD51C* and *RAD51D* are established to increase HGSOC risk. Other germline HR genes might be involved in hereditary OC including *RAD50*, *CHEK2*, *PALB2*, *BARD1*, *NBN* and *MRE11* [22,23]. Lynch syndrome, characterized by germline mutations in mismatch repair genes (*MLH1*, *MSH2*, *MSH6*, *PMS2*) or *EPCAM* (implicated in post-replicative proof-reading and genome integrity) [24], has been identified as responsible for 10–15% of hereditary OC cases, predominantly endometrioid [25,26].

Patients with g*BRCA*m are younger at presentation and diagnosis [27,28]. A meta-analysis showed that women with *BRCA*m (not specified if somatic mutation carriers are included) OC have an increased OS (*BRCA1*: HR 0.76, 95% CI: 0.70–0.83; *BRCA2*: HR: 0.58, 95% CI: 0.50–0.66), regardless of tumour stage, grade, or histologic subtype [29]. However, this remains controversial as a large Canadian series showed that although there is an initial survival advantage among g*BRCA*m carriers, it was not a predictor of long-term survival at 10 years [30]. Although *BRCA1* and *BRCA2* are intimately involved in DNA damage repair; specific roles of *BRCA1* and *BRCA2* are unique and distinct [31]. As a result, published work illustrates potential differences in survival and response to treatment dependent on *BRCA*1 or *BRCA*2 mutation. Several reports have suggested *BRCA2*m patients have a survival advantage and increased response to platinum-based chemotherapy compared to those with *BRCA1*m [32,33,34,35].

### 1.2. Ovarian Cancer Screening and Prevention in BRCA1/2 Mutation Carriers

Screening has been relatively ineffective and no screening strategies in OC have been found to decrease mortality [36,37]. Given the lack of success of screening procedures, risk-reducing salpingo-oophorectomy remain the gold standard [37].

Pharmacological and surgical prevention strategies have been explored in g*BRCA*m carriers. A case-control study found reduced risk of OC in women taking oral contraceptives compared to women who did not (*BRCA1*: odds ratio 0.56, *p* < 0.0001; *BRCA*2: odds ratio 0.39, *p* = 0.0004) [38]. Studies assessing anti-inflammatory drugs such as aspirin in g*BRCA*m carriers are currently ongoing (NCT03480776). Risk-reducing surgical strategies include salpingo-oophorectomy and salpingectomy, which allows the preservation of ovarian function. A prospective observational study showed that oophorectomy or salpingo-oophorectomy reduced the risk of OC by 80% [39]. A Cochrane meta-analysis including ten cohort studies and 8087 *BRCA*m carriers concluded with very-low certainty evidence that risk-reducing sapingo-oophorectomy reduces risk of death from OC [40]. A large cohort study found reduced risk of OC with tubal-ligation in *BRCA*1/2 mutation carriers (HR, 0.43; 95% CI, 0.24–0.75; *p* = 0.003) [41]. A meta-analysis where the prior cohort study was excluded, showed a trend towards reduced risk of OC with tubal-ligation in *BRCA*1 mutation carriers (odds ratio 0.69, *p* = 0.098), but not in *BRCA*2 mutation carriers (odds ratio 0.73, *p* = 0.33), likely due to small number [42]. A proof-of-concept observational study is assessing the safety of salpingectomy with delayed oophorectomy (NCT01907789).

## 2. Treatment of Ovarian Cancer & Implications of *BRCA1/2* Status

### 2.1. Surgery

Primary surgery is one of the mainstream treatments of OC. Optimal cytoreduction in OC cancer is associated with an OS benefit [43]. Retrospective studies have shown that g*BRCA*m-associated and sporadic HGSOC have similar rates of primary optimal debulking [44]; however, those harboring g*BRCA*m have been linked to higher peritoneal tumour load, reduced frequency of ovarian masses and increased bulky lymph nodes at diagnosis [45]. Furthermore, there are morphologic differences (micropapillary architecture and pushing pattern) in the metastases of g*BRCA*m HGSOC with different characteristics of disease spread compared to sporadic HGSOC [46].

At tumour relapse, secondary surgical cytoreduction is recommended in selected *platinum sensitive* recurrences. Selection criteria include optimal primary surgery, absence of ascites, tumour resectability and good performance status [47]. Although there is contradictory data, secondary cytoreduction has been associated with a progression free survival (PFS) benefit when patients are well selected, but no overall survival (OS) benefit has been demonstrated [47,48]. Outcome improvement is linked to the absence of residual disease post debulking surgery. *BRCA1/2* status has not been determined as a factor influencing the outcome of secondary cytoreductive surgery and is not incorporated in the surgical scores to predict an optimal cytoreduction [47,48,49,50,51,52]. g*BRCA*m carriers have also been linked with an increase of visceral metastases at relapse, which makes secondary surgeries more challenging [53].

### 2.2. Intraperitoneal (IP) Chemotherapy

OC tends to metastasize but remains often confined into the surface of the peritoneal cavity. A higher concentration of drug as well as a slower clearance from the peritoneum is achieved with intraperitoneal chemotherapy [54]. Frontline intraperitoneal chemotherapy can be used in stage III HGSOC treatment following surgery, in patients with complete cytoreduction or residual disease <1 cm [55,56,57]. Mature data from over 10 years and three meta-analyses have demonstrated an OS benefit comparing IP to intravenous chemotherapy; although, the debate remains given new options and concerns regarding design of past studies and toxicity [58,59,60,61]. Among patients participating in GOG-172 randomised phase III study aberrant *BRCA1* expression, assessed by immunohistochemistry, was detected in 52% of the women, and correlated with increased OS in those treated with IP chemotherapy vs. intravenous chemotherapy (84 vs. 47 months; *p* = 0.0002) [56,62]. Given the BRCA assessment technique limitations, further studies where *BRCA* sequencing is performed are needed to validate these findings.

More recently, the addition of hyperthermic intraperitoneal chemotherapy (HIPEC) with cisplatin to interval cytoreductive surgery, following 3 cycles of neoadjuvant chemotherapy, was shown to increase recurrence-free survival (10.7 vs. 14.2 months; HR 0.66, *p* = 0.003) and OS (33.9 vs. 45.7 months; HR 0.67, *p* = 0.02) in stage III epithelial OC in a phase III randomised trial. Results remain controversial due to statistical considerations given the small sample size and absence of stratification according to *BRCA1/2* status and histological subtype [63]. Although *BRCA*m was not assessed in this study, there is retrospective data suggesting a possible increase in efficacy in this population [64]. In fact, at the cellular level hyperthermia inhibits HR DNA repair, through degradation of *BRCA2*. Hyperthermia is a potential enhancer of the efficacy of DNA-damaging agents, such as intraperitoneal platinum and poly ADP-ribose polymerase inhibitors (PARPi); although, these findings need to be confirmed [65,66,67]. 

### 2.3. Intravenous (IV) Chemotherapy

#### 2.3.1. Platinum

Platinum chemotherapy remains the backbone of HGSOC treatment. Platinum salts bind to nuclear DNA, forming a variety of structural adducts and trigger cellular responses to DNA damage, replication and transcription inhibition, cell cycle arrest and apoptosis [68,69]. Carboplatin and paclitaxel combination regimen is the well-established standard first-line treatment in advanced OC [70,71] and at the time of relapse for OC patients who are considered *platinum sensitive* [72,73,74].

The impairment of DNA repair processes caused by *BRCA1/2* mutations is a key mechanism involved in the enhanced response to platinum chemotherapy damage [75]. In vitro studies have shown increased sensitivity to platinum agents in cells harboring *BRCA*m, likely due to a loss of *BRCA* mediated DNA damage response and DNA repair [76,77]. A small interfering RNA screen to identify gene enhancers of chemotherapeutic agents showed that silencing of *BRCA*1/2 potentiates cisplatin cytotoxicity [78]. Similarly, an increased sensibility to platinum chemotherapy has been found in *BRCA*m breast and OC xenografts [79,80]. Consistent with these pre-clinical findings, women with g*BRCA*m OC have a higher response rate (RR) to platinum-based chemotherapy. Several series have found that the majority of *BRCA*m women achieve partial or complete responses with platinum agents [81,82,83,84]. Interestingly, even in the setting of platinum resistant disease there may be a role of considering rechallenge with platinum chemotherapy in g*BRCA*m carriers. A case-control study also showed high responses to platinum chemotherapy in *platinum resistant* g*BRCA*m patients, with a CA125 decrease of 80% vs. 43.6%, in comparison to non g*BRCA*m carriers [83]. Patients who are germline *BRCA* wildtype (g*BRCA*wt) but respond to several lines of chemotherapy are more likely to harbor somatic *BRCA*m (s*BRCA*m) [83].

Although the numbers are too small, and direct comparisons between *BRCA1* and *BRCA2* have not been conducted, a 316 patient cohort study suggested that *BRCA2*m has an increased response to first-line chemotherapy in comparison to *BRCA*wt and *BRCA1*m patients [35]. This may be explained by the distinct role of *BRCA2* in DNA repair, regulating RAD51 protein, which is required for ds-DNA break repair [85]. The presence of HR mutations other than *BRCA*, has also been found to be predictive of primary platinum sensitivity [86].

#### 2.3.2. Non Platinum Chemotherapy

##### Taxanes

Taxanes are microtubule stabilizing agents that interfere with the formation of mitotic spindles, causing cell cycle arrest and ultimately apoptosis [87]. For example, paclitaxel is an effective treatment for OC in the first-line in combination with platinum agents and in relapsed settings as monotherapy. A phase III study showed a better outcome with the combination of paclitaxel–cisplatin regimen than for those on a combination with cyclophosphamide [88]. Paclitaxel is also one of the most active non-platinum drugs used as monotherapy in recurrent OC [89,90,91]. The drug can be administered three weekly or weekly, showing a potential ‘anti-angiogenic’ effect when using the weekly regimen [92]. A phase III study comparing three weekly carboplatin and paclitaxel to three weekly carboplatin and dose dense weekly paclitaxel as front line treatment in Japanese women showed improvement in progression free survival and overall survival with the combination dose dense regimen (PFS HR 0.76, *p* = 0.0037; OS HR 0.79, *p* = 0.039), which was not confirmed in European studies (MITO-7 three weekly vs. weekly carboplatin and paclitaxel PFS: HR 0.96, *p* = 0.66; ICON-8 three weekly carboplatin and paclitaxel (arm1) vs. three weekly carboplatin and weekly paclitaxel (arm2) vs. weekly carboplatin and paclitaxel (arm 3) PFS: arm 2 vs. 1 HR 0.92 *p* = 0.45, arm 3 vs. 1 HR 0.94, *p* = 0.56), though one of these (MITO-7 trial) reduced dose density of paclitaxel and also gave carboplatin on a weekly basis [93,94,95]. Similarly, another European randomised phase III study on paclitaxel monotherapy failed to note any differences in the two administration types [96]; however, the dose dense regimen is well established in *platinum resistant* recurrent disease. To date, there remains no available clinical data regarding the implication of *BRCA*m in dose dense delivery of taxanes.

Inhibition of *BRCA1* expression in OC cell lines has been shown to reduce anti-tumour activity of taxanes, due to differential regulation of apoptosis by BRCA1 protein [76]. The effect of BRCA2 in taxane resistance is less studied. A cell-line study on *BRCA2m* and *TP53*wt OC showed high sensitivity to taxanes and platinum agents [77] although the clinical relevance of these findings is unclear and with limited retrospective data available suggesting no difference in response according to *BRCA1/2* status [97]. Retrospective studies do suggest lower response to taxanes in s*BRCA2m* prostate, and *BRCA1m* breast cancer patients [98,99]. The relationship between taxane sensitivity and *BRCA*m in OC requires further investigation. 

##### Trabectedin

Trabectedin is a DNA alkylating agent that binds to the DNA minor groove and modifies the tumour microenvironment by reducing the pro-inflammatory mediators. The sensitivity to trabectedin is linked to an efficient transcription-coupled nucleotide excision repair and deficient HR repair activity in the target tumour cells [100,101].

A phase III study including *platinum sensitive* and *resistant* patients, showed PFS improvement (7.3 vs. 5.8 months; HR 0.79 *p* = 0.019), without OS benefit for the combination of trabectedin and pegylated liposomal doxorubicin (PLD), versus single agent PLD [102]. Subgroup analyses suggested a trend towards OS benefit in the *platinum sensitive* cohort (HR 0.85, *p* = 0.092), which was particularly enhanced in the partial *platinum sensitive* population with a PFI of 6–12 months (23 vs. 17.1 months; HR 0.59 *p* = 0.0015). This finding led the European Medicines Agency (EMA) to approve trabectedin combined with PLD in patients with relapsed, *platinum sensitive* OC [103,104]. Subgroup analyses also showed that the combination had a better response in g*BRCA1*m carriers [105]. The results of a phase III study comparing the experimental arm of PLD with trabectedin versus PLD with carboplatin in partial *platinum sensitive* OC (NCT01379989) are pending.

A single-arm phase II study in patients with *BRCA*m or *BRCA*ness phenotype (repeated platinum-sensibility), showed high RR to trabectedin (Overall response rate (ORR) 39.4%; *platinum sensitive* 47.8%, *platinum resistant* 31.2%), with no statistically significant difference according to the mutational status [106].

##### Pegylated Liposomal Doxorubicin (PLD)

Doxorubicin is a cytotoxic anthracycline that produces DNA damage, inhibiting DNA topoisomerase II. The topoisomerase II-doxorubicin-DNA complex has been shown to induce dsDNA breaks. In the case of PLD, the pegylated liposomal coating provides stabilization in blood as well as increased blood circulation time, achieving increased accumulation in tumours with high vascular permeability [107].

PLD has activity in OC, when used in combination with carboplatin in the setting of *platinum sensitive* recurrence [108], or as monotherapy in *platinum resistant* disease [109,110]. The combination of carboplatin and PLD failed to show superiority in comparison to carboplatin and paclitaxel in first-line setting, but it did show equal efficacy in platinum sensitive recurrent disease with a favorable toxicity profile [111,112].

Given the mechanism of effect of PLD in DNA repair, *BRCA*m OC might have an increased sensitivity to this agent. Retrospective data shows increased response to PLD in patients with *BRCA*m [97,113,114]. A randomised phase II study on *gBRCA*m OC patients who recurred prior to 12 months since the last platinum-based chemotherapy, compared PLD to single agent olaparib and showed no differences in PFS (HR 0.88, *p* = 0.66) [115]. This was one of the first trials that restricted eligibility to women with g*BRCA*m ovarian cancer, with partially platinum sensitive and platinum resistant disease; as a consequence, the median PFS of the PLD arm was higher than had been predicted at 7.1 months, and explained as perhaps an non-anticipated consequence of the enrichment strategy, due to exclusion of women without g*BRCA*m and other histologic subtypes which are more resistant (clear cell, mucinous) [109,110,115].

### 2.4. Targeted Treatment

#### 2.4.1. PARP inhibitor (PARPi)

Poly(ADP-ribose) polymerases (PARPs) are enzymes that transfer ADP-ribose groups to target proteins. They function in cellular signaling pathways, including DNA repair, transcriptional regulation, RNA interference, mitochondrial function, formation of subnuclear bodies and in cell division [116]. In humans, there are 17 members of the *PARP* gene family, with PARP-1 and PARP-2 being the most studied due to their involvement in DNA damage detection and repair [117]. As a result of DNA single strand breaks (SSBs), a process called PARylation occurs whereby PARP-1 and PARP-2 produce large branched chains of ADP-ribose polymers from nicotinamide adenine dinucleotide (NAD+). Subsequently these polymers directly participate or coordinate the repair process, including the base excision repair (BER) [118].

**Figure 1:**
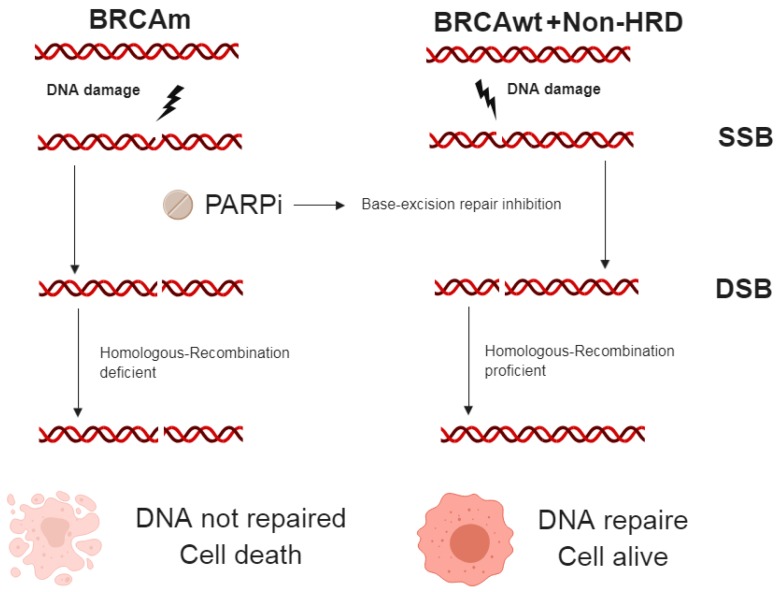
Synthetic lethality concept [119]. A DNA damage can lead to a single-strand break (SSB), and PARP enzymes play an essential role in SSB repair. The administration of PARPi will inhibit base-excision repair for SSB. If SSB are not repaired, continuous damage can progress to double-strand breaks (DSB). DSBs are usually repaired through the *BRCA* dependent homologous-recombination process. However, in cells with *BRCA*m the homologous-recombination pathway for DSB repair is damaged and leads to cell death. *BRCA*m: *BRCA* mutation, *BRCA*wt: *BRCA* wildtype, Non-HRD: No homologous recombination deficiency, SSB: single strand break, DSB: double strand break.

The rationale for the development of PARP inhibitors (PARPi) was based on the concept of synthetic lethality, whereby mutations in two genes are synthetic lethal, but a mutation of one alone maintains viability of cells (Figure 1) [119]. PARP1 is involved in the SSBs but is not a major determinant in dsDNA repairs [120]; although a persistent damage in SSBs may cause the collapse of the replication forks, producing DSBs, which are commonly repaired by HR enzymes [121]. Thus, cancer cells with *BRCA*m treated with PARPi accumulate SSBs, leading to DSBs that are not repaired by the HR pathway, ultimately resulting in cell cycle arrest or death [122].

PARPi have been evaluated as single-agents, in combination and as maintenance treatment following response to platinum-based chemotherapy in OC. Olaparib is the most investigated PARPi and the first agent approved by the European and American regulatory agencies. Several PARPi have now also been approved due to level 1 evidence in ovarian cancer including niraparib and rucaparib. Talazoparib was recently approved by the U.S. Food and Drug Administration (FDA) for g*BRCA*, HER2 negative breast cancer.

##### Single Agent Treatment

Several phase I and II trials have been published on single agent PARPi focusing on *BRCA*m population (Table 1). Gelmon et al. demonstrated activity of olaparib in women without *BRCA*m and the influence of platinum sensitivity or resistance. Kaufman et al. showed in a phase II trial that olaparib is effective as treatment across different tumour types with g*BRCA1*/2m. In the OC cohort, 193 heavily pretreated women, either *platinum resistant* or non-suitable for platinum, were included, showing an ORR of 31.1% [123,124]. This study led to the FDA approval of olaparib in *BRCA*m OC treated with three or more lines of chemotherapy [125].

Data across different PARPi used as single agent suggest that RR are higher in *BRCA*m population. In Part 1 of ARIEL2 (NCT01891344) clinical trial, women with recurrent platinum sensitive HGSOC were treated with rucaparib. A tumour-based molecular signature of HR deficiency (HRD) was developed, classifying patients according to *BRCA*m and loss of heterozygosity (LOH), representing HRD. PFS was significantly higher in the *BRCAm* (HR 0.27, *p* < 0.0001) and LOH-high (HR 0.62, *p* = 0.011) compared to LOH-low subgroup [126]. Data also suggests a higher activity of single agent PARPi in *platinum sensitive* population (Table 1) [127,128,129,130,131,132,133].

The activity of single agent PARPi has been compared to chemotherapy in a randomised phase II trial in women with platinum resistant and partially platinum sensitive disease, comparing two doses of olaparib with PLD in g*BRCAm* OC, showing no statistical significant differences in PFS (HR 0.88, *p* = 0.66) [115].

##### Maintenance Treatment

PARPi have been assessed as maintenance treatment in frontline and in *platinum sensitive* recurrence (Table 2), following response to platinum chemotherapy.

##### First Line

**Figure 2:**
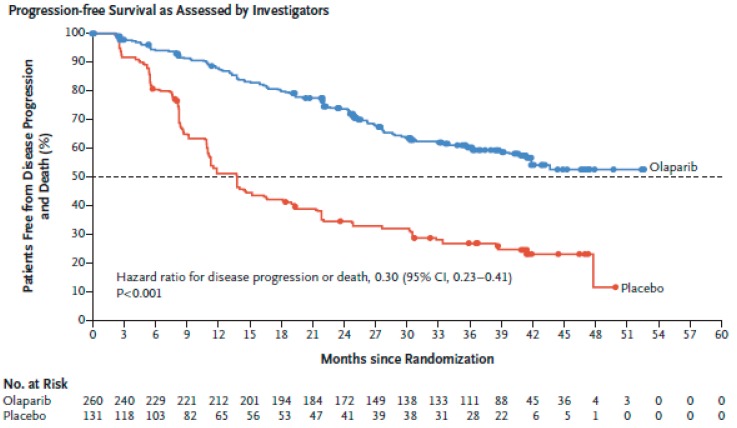
Kaplan–Meier estimates of the rate of freedom from disease progression, as assessed by investigators, and from death in the olaparib group and the placebo group of SOLO1 clinical trial.

The phase III SOLO1 (NCT01844986) trial assessed olaparib/placebo maintenance treatment in stage III and IV OC with germline or somatic *BRCA*m, following response to first-line platinum-based chemotherapy [139]. The s*BRCA*m population in this study was underrepresented (two centrally confirmed s*BRCA*m of the 391 patients). The risk of disease progression at three years was significantly lower in the olaparib arm (60% vs. 27%; HR 0.3, *p* < 0.001) (Figure 2). Time from randomisation to progression on second line of treatment (PFS2) at three years was superior in the olaparib arm (HR 0.5, *p* < 0.001). Overall survival data continues to be immature but no significant differences have been found so far (Table 2). Other studies assessing frontline maintenance PARPi treatment non-restricted to *BRCA*m carriers are ongoing (PRIMA, NCT02655016).

##### Platinum Sensitive Relapse

Results of Study19 (NCT00753545) lead to the approval of olaparib as the first PARPi as maintenance treatment for *BRCAm* OC by the European Medicine Agency (EMA). It was subsequently approved in the maintenance setting by the FDA and Health Canada. 

Study19 was a phase II placebo-controlled trial of *platinum sensitive* OC treated with at least two prior lines of platinum-based chemotherapy and response to the last treatment. There was a significantly longer PFS in the olaparib arm (8.4 vs. 4.8 months; HR 0.35, *p* < 0.001) [140]. A preplanned retrospective analysis of the data by *BRCA*m status identified 51% of patients harbored a germline or somatic *BRCA*m, being associated with a significant PFS increase in the olaparib group (11.2 vs. 4.3 months; HR 0.18, *p* < 0.0001); *BRCA*wt women also had PFS improvement; however, the difference between the groups was lower (7.4 vs. 5.5 months; HR 0.54, *p* = 0.0075). Somatic *BRCA*m were found in 14% of women, having fewer progression events when treated with olaparib (38% vs. 60%) [141]. Although the study was not powered to assess OS, no statistically significant differences were found (HR 0.73, 95% CI 0.55–0.96; *p* = 0.025, required threshold *p* < 0.0095). [142].

SOLO2/ENGOT-Ov21 (NCT01874353) phase III randomised trial assessing olaparib/placebo as maintenance treatment in *platinum sensitive* OC with g*BRCA*m (s*BRCA*m not included), showed that the investigator-assessed PFS was significantly longer in the olaparib arm (19.1 vs. 5.5 months; HR 0.3, *p* < 0.0001). OS data was still immature (HR 0.8, *p* = 0.43) [143].

Rucaparib and niraparib have been also assessed as maintenance treatments in randomised double-blind phase III trials, but the population included in these studies was not limited to those carrying *BRCAm*. The ENGOT-OV16/NOVA (NCT01847274) trial, assessing niraparib maintenance, included two independent cohorts, g*BRCA*m and non-g*BRCA*. Tumour testing was performed on archival tissue to define HRD; 553 patients were enrolled, of whom 37% harbored g*BRCA*m. PFS was significantly longer in the niraparib arm in all pre-specified cohorts, g*BRCA*m (21 vs. 5.5 months; HR 0.27, *p* < 0.001), non-g*BRCA*m with HRD (12.9 vs. 3.8 months; HR 0.38, *p* < 0.001), and *BRCA*wt subgroup (9.3 vs. 3.9 months; HR0.45, *p* < 0.001). Those with s*BRCA*m had a similar reduction in the risk of progression than g*BRCA*m. Niraparib also showed PFS improvement in the HRD-negative exploratory subgroup (6.9 m vs. 3.8 m; HR 0.58, *p* = 0.02) [144].

The randomised phase III trial, ARIEL3 (NCT01968213), assessing rucaparib maintenance therapy following completion of platinum-based chemotherapy for platinum sensitive recurrence used the same LOH assay as described in ARIEL2 part1, with a modified cut off point refined from Ariel 2, to validate *BRCA*m and LOH as potential biomarkers. Rucaparib maintenance improved PFS in all pre-specified subgroups, germline and somatic *BRCA*m carriers (16.6 vs. 5.4 months; HR 0.23, *p* < 0.0001), HRD tumours, including *BRCA*m and LOH-high (13.6 vs. 5.4 months, HR 0.32, *p* < 0.0001), and in the intention-to-treat population (10.8 vs. 5.4 months, HR 0.36, *p* < 0.0001). Interestingly, in the exploratory non-HRD subgroup rucaparib also demonstrated benefit (HR 0.58, *p* = 0.0049) [145].

There are several notable differences in the three PARPi maintenance phase III trials described above, which may influence data interpretation. Regarding the clinical parameters, NOVA trial was restricted to patients with residual disease <2 cm, while the other trials did not have disease size restriction. The inclusion criteria for Cancer antigen 125 (CA-125) levels was also different in the three trials; whereas ARIEL3 was restricted to those with a normal level, NOVA included those with a decrease greater than 90% in the last platinum-based regimen, and SOLO2 was less restrictive allowing all patients without an evidence of rising CA-125. SOLO2 only enrolled women with a g*BRCA*m, while the trials assessing niraparib and rucaparib also enrolled patients without *BRCA*m. In addition, the subgroup classification was different, s*BRCA*m patients were analysed as part of the HRD subgroup in NOVA, whereas in ARIEL3, s*BRCA*m patients were part of the *BRCA*m subgroup. Different assays were used in order to define the HRD subgroup; NOVA applied the Myriad mychoice HRD test and ARIEL3 the Foundation medicine T5 NGStool [143,144,145]. Toxicity data along the different PARPi are summarized in Table 3.

Although a longer PFS was reported for PARPi maintenance in women with *BRCAm* and HRD, the assays were unable to accurately predict who would not benefit from PARPi in NOVA, ARIEL3 and STUDY19 trials. These studies have also shown improvement in time to second subsequent treatment/ PFS2; however, OS data are still immature [140,141,142,143,144,145]. PARPi treatment is also associated to high drug acquisition cost [148]. Some women on PARPi have durable long-term control of disease, in some cases approaching 10 years. Learning from these long term PARPi responders may provide valuable information and improve our understanding of the associated mechanisms of action and define predictive biomarkers [140,149,150,151]. A study on long term responders with >2 years of olaparib response is ongoing (OLALA, NCT02489058).

##### Combination Treatments

Several PARPi have been assessed in combination with chemotherapy, anti-angiogenics, immunotherapy or targeted treatments. Contemporary combination trials have not been restricted to *BRCAm* population.

The combination of chemotherapy and PARPi was assessed in STUDY41 (NCT01081951), a randomised phase II trial where patients with *platinum sensitive* recurrence received either olaparib in combination with carboplatin and paclitaxel followed by maintenance olaparib, or carboplatin and paclitaxel alone. Women receiving olaparib had an improvement in PFS (12.2 vs. 9.6 months; HR 0.51, *p* = 0.012), with a greater benefit seen in *BRCA*m patients (HR 0.21, *p* = 0.0015). Although the study was not designed to measure the contribution of each treatment, the late separation of the PFS curves suggested that the maintenance phase was the key contributor to the improvement [149].

The combination of PARPi with PLD has also been explored in a phase I dose-finding trial with 44 patients, using continuous or intermittent schedule. In this trial, 28 OC patients were included, from which 11 g*BRCA*m, with an ORR 50% and 61% respectively [152]. The maximum tolerated dose was not reached using continuous olaparib 400 mg twice daily and PLD (40 mg/m^2^). Currently a single arm phase II trial is ongoing in *platinum resistant* OC irrespective of the *BRCA*m status, assessing the combination of PLD 40 mg/m^2^ every 28 days and olaparib 300 mg twice daily (NCT03161132).

Antiangiogenic treatment has also been explored in combination with PARPi. Preclinical studies have shown a synergy between the two pathways. In fact, antiangiogenics can induce tumour hypoxia, leading to downregulation of *BRCA* and *RAD51*, and possibly increasing tumour sensitivity to chemotherapy and PARPi [153,154,155]. Preclinical data demonstrated that hypoxia-mediated defects in DNA repair can lead to genetic instability and drive oncogenesis [156]. In a randomised phase II study olaparib was compared to the combination of olaparib and cediranib, a vascular endothelial growing factor (VEGF) receptor and c-kit tyrosine kinases inhibitor, in patients with *platinum sensitive* HGSOC, endometrioid or g*BRCA*m OC. There was a PFS (17.7 vs. 9 m, HR0.42, *p* = 0.005) and ORR benefit (56% vs. 84%, *p* = 0.008) in the combination arm. Interestingly, in subgroup analysis the benefit was only significant in g*BRCA*wt or unknown status (HR 0.32, *p* = 0.008), with a trend in g*BRCA*m subgroup (HR 0.55, *p* = 0.16). Although the study was not powered to detect OS differences, updated results have found a trend towards OS improvement in *BRCA*wt (HR 0.48, *p* = 0.074). Drug-related adverse events (AEs) were more common in the combination arm, with 70% experiencing grade 3 or higher events [157,158]. A non-randomised phase II study with olaparib and cediranib combination in the cohort of *platinum sensitive* and *resistant* populations showed an ORR of 77% and 20%, respectively. Responses were seen in *platinum resistant BRCA*wt women, but benefit was higher in those with g*BRCA*m. [159]. In *platinum sensitive* population, the OVC1 (NCT02446600) phase III trial assessed olaparib vs. olaparib and cediranib vs. platinum-based chemotherapy; results are awaited. Similarly, OVC2 (NCT02502266) is a randomised trial assessing the combination in *platinum resistant* setting and currently ongoing. Other combinations with bevacizumab, an anti-VEGF monoclonal antibody, are also being assessed (PAOLA1, NCT02477644; AVANOVA, NCT02354131; OVARIO, NCT0332619).

PARPi have been found to induce PI3K pathway activity in preclinical models, whereas PI3K suppression induces DNA damage [160,161,162]. Combination of a PI3K inhibitor with PARPi has shown synergy in *BRCA1*m and *BRCA*wt breast cancer xenografts [161]. A phase I trial assessing BKM120, a PI3K inhibitor, and olaparib included 46 OC patients, showing an ORR of 29% (90% CI: 18–43%) irrespective of platinum-sensitivity. Dose limiting toxicities included grade 3 transaminase elevation and depression [163]. Similarly, a phase I trial combining olaparib and vistusertib, a mTORC1/2 inhibitor, has shown an ORR of 20% in the OC subgroup, where most of the patients were *platinum resistant* [164]. The combination of olaparib and AZD5363, AKT inhibitor, has also been assessed in a phase I multitumour study, showing an ORR of 24% [165]. Further studies are needed on this combination for *BRCA*m and wild-type patients.

The best treatment option at the time of PARPi progression remains unclear; although, retrospective data suggests that there is no significant clinical cross-resistance between PARPi and platinum-based chemotherapy and platinum continues to be the treatment of choice [166]. Whether retreatment with PARPi or new combinations are beneficial, especially in those harbouring *BRCA*m or HRD, is under investigation (NCT03106987, NCT02340611).

#### 2.4.2. Antiangiogenic Treatment

Angiogenesis is one of the hallmarks in OC pathogenesis. VEGF and angiogenesis promote tumour growth and progression, as well as ascites formation, and vascular density is a negative prognostic marker [167,168,169,170]. Anti-angiogenic agents are part of the OC treatment in frontline as well as in the relapsed setting, regardless of BRCAm status. HRD or BRCAm status has not been found to be a response biomarker [171,172] for antiangiogenics.

In the frontline setting, addition of bevacizumab to carboplatin and paclitaxel, followed by maintenance bevacizumab has shown a PFS benefit in ICON7 (NCT00483782) (for an additional 12 cycles at 7.5 mg/kg; HR 0.81 *p* = 0.004) and GOG-218 (NCT00262847) (for a total of 22 cycles at 15 mg/kg; HR 0.7 *P* < 0.001) randomised phase III studies. No OS benefit was found in the intention-to-treat population [173,174]. However, high-risk subgroup in an exploratory analysis of ICON7 (suboptimally debulked stage III/IV, non-operated patients) had a significant OS improvement (HR 0.78 *p* = 0.03) [175]. The benefit of anti-angiogenic agents frontline was further assessed with pazopanib, showing PFS (17.9 vs. 12.3 months; HR 0.77, *p* = 0.0021) but no OS benefit. Toxicity was challenging, with 33.3% treatment discontinuations [176]. g*BRCAm* status has been assessed retrospectively and is not a predictive biomarker for antiangiogenic combination therapy [172].

#### 2.4.3. Immunotherapy

Preclinical data has shown that intact immune surveillance is needed to control tumour transformation and growth [177]. In fact, restoring anti-cancer immunity by immune checkpoint blockade has been successful in several tumours. The activation of immune response requires the recognition of the cancer antigen by T cell receptors (TCR), as well as several co-stimulatory and inhibitory signals [178,179]. PD1 and its receptor PD-L1 play an important role in the interaction of tumour cells and tumour-specific T cells [180]; antibodies against these immune checkpoints can activate the anti-tumour response.

HGSOC has a relatively low tumour mutational burden (TMB) and immunogenicity, with low PDL1 expression in late progressing tumours [181]. However, cancer cells with genomic instability and deficiency in DNA repair, such as microsatellite unstable or *POLE* mutated tumours have a higher TMB, source of neoantigens, and thus an increased response to immune checkpoint inhibitors [182,183]. Similarly, germline and somatic *BRCA*m HGSOC have a higher TMB, as well as increased immune infiltrates, which are not seen in HR proficient HGSOC [184,185]. Tumour-infiltrating T cells are also increased in *BRCA1*m OC [186]. Thus, *BRCA*m HGSOC might have an immunogenic phenotype, and be a potential biomarker for treatment with immunotherapy [181].

Several antibodies against PD-1, PD-L1, CTLA4 have been tested in OC patients, most of them without any selection biomarker. Multi-tumour or small early phase trials have shown modest activity of single agent immunotherapy with ORR that ranges between 6% and 11.5% [187,188,189,190]. KEYNOTE-100 study (NCT02674061), a phase II single-arm trial assessing pembrolizumab in recurrent OC included two cohorts: cohort A with up to three lines of treatment, and cohort B with 4–6 prior lines of treatment. The study showed a modest anti-tumour effect, with ORR of 8%, 7.4% and 9.9%, for all patients, cohort A and B, respectively. Response according to histological subtypes or *BRCA*m status has not been reported [191]. Other immunotherapy agents such as indoleamine 2,3-dioxygenase-1 (IDO1) inhibitor and CA125 targeting vaccines have shown no significant efficacy in OC [192,193].

Given the modest results of single agent immunotherapy, there is an effort to find combinations that could overcome resistance to checkpoint inhibitors and increase immunogenicity of OC. Several on-going trials are combining checkpoint inhibitors with chemotherapy, PARPi or vaccines (NCT03330405, NCT03029403). 

Preclinical models indicate synergy between PARPi and checkpoint inhibitors, regardless of *BRCA*m or PDL1 status [194,195,196]. A phase I-II trial, TOPACIO (NCT02657889), on recurrent OC (*platinum sensitive* 17%; *BRCA*wt 77%) assessing the combination of niraparib and pembrolizumab, has shown an ORR of 25%, with duration of response of 9.3 months. In *platinum resistant* patients, the efficacy was found to be similar regardless of *BRCA*m and HRD [197]. MEDIOLA (NCT02734004) assessing the combination of olaparib and durvalumab showed an ORR of 63% in the g*BRCA*m *platinum sensitive* OC cohort [198].

Combination trials are ongoing in frontline and recurrent setting, where immune checkpoint inhibitors are combined with chemotherapy, antiangiogenics and PARPi.

#### 2.4.4. Other Agents Under Investigation

##### Cell Cycle Modulators

Cell cycle checkpoints are essential for DNA repair and maintenance of genomic integrity. WEE-1 kinase is a G2 cell-cycle checkpoint regulator that is activated in response to DNA damage. Thus, WEE-1 inhibitors can abrogate G2 cell cycle arrest. Loss of cell cycle dependent genes and replication stress are being studied as biomarkers of response to this treatment [199]. Most HGSOC are TP53 deficient, having a defective G1 checkpoint [200]. Tumour cells with deficient G1 checkpoint, treated with a WEE-1 inhibitor lose the ability to arrest the cell cycle in response to DNA damage, and enhance apoptosis. Preclinical data have shown potential effectiveness of the treatment in OC xenografts, irrespective of TP53 status and *BRCA1*m [201]. However, a phase I dose-finding multi-tumour study did suggest that *BRCA*m and HRD might be response biomarkers to WEE1 kinase inhibitor monotherapy [202]. Indeed, CDK1, the main target of WEE1, plays a key role in HR and dsDNA break repair [203]. In vitro studies have shown synergy between CDK1 inhibition and PARPi, a combination that is being further investigated [204,205].

A phase II proof-of-concept trial was conducted assessing the combination of WEE-1 inhibitor, AZD1775, as an enhancer of carboplatin on *TP53* mutated *platinum resistant* (PFI ≤ 3 m) OC upon first recurrence. An ORR of 43% was found, suggesting a possible synergy. There were two prolonged response patients, one of them harbored a *BRCA1*, *MYC* and CCNE1 alterations [206].

Prexasertib, a checkpoint kinase 1 and 2 inhibitor, has been assessed in a proof-of-concept phase II study in recurrent HGSOC, showing an ORR of 29% in non-g*BRCA*m carriers, results awaited for the g*BRCA*m cohort [207].

## 3. *BRCA* Mutations and Treatment Resistance

The impact of *BRCA*m on treatment response might be variable, as *BRCA*m in the tumour might evolve over time as resistance develops. Secondary point mutations that are actionable are rare in relapsed chemotherapy resistant HGSOC [208]. Several mechanisms of resistance to PARPi and platinum have been reported, including increased HR capacity with *BRCA* reversions mutations, non-homologous end-joint (NHEJ) repair alterations, reduction in PARP1 activity and cell stromal alterations, such as changes in cellular adhesion molecules [208]. Mutations in the RING-type zinc finger (RING) domain of *BRCA1* have also shown to be predictive of poor response to platinum and PARPi [209].

### 3.1. Increased HR Activity

**Figure 3:**
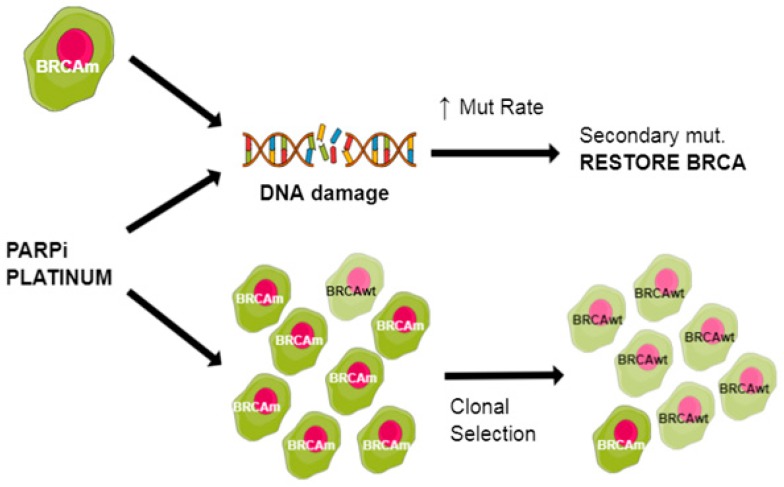
Potential mechanisms to restore BRCA function.

Secondary *BRCA*m that restore BRCA1/2 function are the most studied mechanism of drug resistance to platinum and PARPi [208,210,211]. However, the mechanism of acquiring drug resistance is still under investigation. DNA damaging agents or BRCA1/2 deficiency itself exhorts selective pressure, leading to adoptive changes and increased mutational rate. Tumours are heterogeneous and clonal selection after treatment with DNA damaging agents (Figure 3) may select for resistance [212]. 

Tumour heterogeneity has been found across different studies. Patch et al. performed germline and somatic whole genome sequencing in 92 OC and matched with acquired resistant disease. In five cases reversion mutations were found, out of which two cases contained multiple mutations. An autopsy revealed 12 different reversion *BRCA2*m [208]. Similarly, 12 patients with tissue post-progression on rucaparib from ARIEL2 study found *BRCA1*, *RAD51C* and *RAD51D* mutations in six patients, and in five of them one or more secondary mutation restoring the reading frame were seen [213]. Complementary in vitro assays and patient derived xenografts confirmed that resistance to rucaparib was associated with the secondary mutations. Loss of *BRCA1* promoter methylation was also reported as a mechanism to restore BRCA1 function [213]. In addition, methylation of all BRCA1 copies was found to be a predictor of response to rucaparib using patient derived xenografts, while heterozygous methylation was associated with resistance [214].

### 3.2. Non-Homologous End Joining (NHEJ) Factors and Shieldin Complex

In addition to HR, DSB can be repaired by NHEJ through direct joining across the break sites; although, data suggests that HRD cells with disabling NHEJ reverses genomic instability induced by PARPi and rescues from lethality of PARPi [215].

In *BRCA1* deficient cell DNA breaks are aberrantly joined by NEHJ factors, such as 53BP1 and Ligase 4. Interestingly, in *BRCA1* (but not *BRCA2*) deficient cells, the loss of 53BP1 rescues Rad51-dependent HR, partially blocking ATM resection [216,217,218]. Thus, in the absence of both 53BP1 and *BRCA1*, proteins involved in HR are loaded normally at the chromosomal break site, permitting error-free DNA repair. In fact, *BRCA1*/53BP1 double-deficient cells are resistant to PARPi, and combination of both have been suggested as predictive biomarkers of sensitivity [216,217,218]. Decreased 53BP1 expression has not been found to correlate with platinum resistance [219].

Shieldin complex is involved in dsDNA break localization, and its loss impairs NHEJ. Mutations in genes related to shieldin complex can cause PARPi resistance in BRCA1 deficient tumours [220,221]. 

### 3.3. Drug Efflux Pump

Overexpression of drug efflux transporters, particularly ATP-binding cassette membrane transporters, represents an additional mechanism of acquired drug resistance. The *ABCB1* gene encodes the multidrug-resistant protein 1 (MDR1), which is associated with a rapid efflux of many chemotherapeutic agents including paclitaxel, etoposide and doxorubicin. Patch et al. found overexpression of MDR1 activity in 8% of recurrent HGSOC samples analyzed; the biological mechanism involved promoter fusion and translocation enabling overexpression [208]. Evidence suggests that drug efflux pumps might also be involved in PARPi resistance, and clinical trials are being planned to address this mechanism of resistance [222]. 

### 3.4. Stromal Reactions

From the autopsy of a chemo-resistant OC patient, Patch et al. described no reverse *BRCA*m and maintained HR, but an extensive desmoplatic stromal reaction in histological examination [208]. Stromal reactions are associated with poor drug uptake and have been found as chemoresistance mechanisms in other cancers [208,223].

## 4. Conclusions

The presence of germline and somatic *BRCA*m confers distinct characteristics to OC and is now considered to be essential information to plan OC treatment. Assessment of g*BRCA*m is not only a tool for cancer prevention and family counselling, but also a predictive factor confining increased response to DNA damaging agents, such as platinum chemotherapy. PARPi have shown to be effective as single agent and as maintenance treatment, particularly in germline and somatic *BRCA*m carriers. This has led to a major emphasis on *BRCAm* testing. Germline testing was initially only recommended in patients with positive family history; it is now recommended for all HGSOC. Contemporary somatic testing also provides valuable information and impacts patient care. Therefore, *BRCA* reflex tumour testing should be performed at the time of initial diagnosis.

Several trials are ongoing assessing the combination of chemotherapy, immunotherapy and targeted agents in order to overcome resistance. Germline and somatic *BRCA*m should be integrated into routine clinical care and considered as stratification factor in clinical trials given its predictive value. It is also important to understand better resistance mechanisms of *BRCA*m OC, in order to develop more effective and less toxic treatments, maintaining quality of life while and striving to improve survival. Cost-effectiveness of both the new treatments and genetic testing needs to be taken into account when setting priorities in health care policies.

## Figures and Tables

**Table 1:** Phase 1–2 trials assessing single agent PARP inhibitors. N: number of patients. NR: Not reported. PlatS: platinum sensitive. PlatR: platinum resistant. BID: twice daily. QD: Once daily. LOHh: High loss of heterozygosity, LOHl: Low loss of heterozygosity; LOHu: Unkown loss of heterozygosity. MMC: MitomicinC. IV: intra venous. PFI: platinum free interval.

	Phase	N (Ovarian)	Prior Treatments	*BRCA/HR*	Platinum Sensitivity	Dosage	RR (RECIST 1.1) % CI 95%
**OLAPARIB**							
Fong et al. [127] (NCT00516373)	Phase 1	50	1: 10% 2: 22% 3: 22% ≥4: 46%	48 g*BRCA*m (96%)	13 PlatS (27%)	Escalation: 40 mg qd- 600 mg bid. Expansion: 200 mg bid.	28% (16.2–42.5) PlatS: 46.2% (19.2–74.9) PlatR: 33.5% (15.6–55.3) Plat Refractory: 0% (0–24.7)
Mateo et al. [134] (NCT00777582)	Phase 1 Multitumour	Group 1 and 6 (OC): 53	Group 1: median 6 (200 mg bid) and 3 (400 mg bid) Group 6: median 4 (300 mg bid) and 3 (400 mg bid)	Group 1 and 6: 53 g*BRCA*m (100%)	Group 1: NR Group 6: 15 PlatS (37%)	200–400 mg bid.	Group 1 and 6: 30% (18.3–44.3)
Audeh et al. [129] (NCT00494442)	Phase 2	57	Median 3 (400 mg bid) and 4 (100 mg bid)	57 g*BRCA*m (100%)	19 PlatS (33%)	400 mg bid	33% (20–51) PlatS: 38% PlatR: 30%
						100 mg bid	13% (4–31) PlatS: 50% PltR: NR
Gelmon et al. [128] (NCT00679783)	Phase 2 Ovarian and breast cancer	64	Median 3	17 *BRCA*m (26%)	25 PlatS (38.5%)	400 mg bid	29% (19–41) *BRCA*m: PlatS: 60% PlatR:33% *BRCA*wt/u: PlatS: 50% PlatR: 4%
Kaufman et al. [123] (NCT01078662)	Phase 2. Multitumour	193	Mean 4.3	193 *BRCA*m (100%)	All PlatR or intolerant.	400 mg bid	31.1% (24.6–38.1%)
Kaye et al. [115] (NCT00628251)	Phase 2. Olaparib vs. PLD	97	Olaparib 200 mg: ≥3 lines: 59%	97 *BRCA*m (100%)	48 PlatS	200 mg bid	25%
			Olaparib 400 mg: ≥3 lines: 78%			400 mg bid	31%
			PLD: ≥3 lines: 51%			PLD	18%
**NIRAPARIB**							
Sandhu et al. [130] (NCT00749502)	Phase 1	49	Overall population median: 5	22 *BRCA*m (45%)	13 PlatS (27%)	Part A: 30–400 mg qd Part B: 300 mg qd	*BRCA*m: 40% (19–64) PlatS: 50% (19–81) PlatR: 33% (7–70) *BRCA*wt: NR PlatS: 33% (1–91) PlatR: 5% (<1–26)
Moore et al. [135] QUADRA (NCT02354586) Abstract	Phase 2	463	All patients ≥3 prior lines	NR	162 Plat refractory 152 PlatR 118 PlatS 31 U	300 mg qd	HRD: PlatS 27.5% (15.9–41.7) *BRCA*m: 38.9% (7–18) *BRCA*wt: 21.2% (7–33) Non-HRD: NR.
**RUCAPARIB**							
Drew et al. [136] (NCT00664781)	Phase 2. OC and breast cancer	51	Overall population: median 2	47 *BRCA*m (92%)	NR	IV (4–18 mg/m^2^, 5 days q 3w) Oral (92 qd–600 bid).	Overall population OC + breast: 7%. IV cohort: 2% Oral cohort: 15%
Kristeleit et al. [137] STUDY 10 (NCT01482715)	Phase 1–2. Multitumour	Part 1: 20 OC (multi-tumour)	Overall population: Median 4	Part 1: Overall population 64%	Part 1: Overall population 14.3%	Part 1: 40 mg qd–840 mg bid	Part 1: NR
		Part 2A: 42 (only ovarian)	Median 2	Part 2A: 42 *BRC*Am (100%)	Part 2A: 42 PlatS. (100%)	Part 2A: 600 mg bid.	Part 2A: 59.5% (43.3–74.4)
Swisher et al. [126] ARIEL 2 part 1 (NCT01891344)	Phase 2	206	*BRCA*m: ≥2 lines 58% LOHh, *BRCA*wt: ≥2 lines 46% LOHl, *BRCA*wt: ≥2 lines 33% LOHu, *BRCA*wt: ≥2 lines 17%	40 *BRCA*m (19%) 82 LOHh (*BRCA*wt) 70 LOHl (*BRCA*wt) 12 LOHu (*BRCA*wt)	206 PlatS (100%)	600 mg bid	*BRCA*m: 80% (64–91) LOHh: 29% (20–40) LOHl: 10% (4–20) LOHu: 33% (10–65)
Oza et al. [131] Pool data: ARIEL2+ STUDY 10	Phase 2	106 (integrated efficacy population)	≥3 prior chemotherapies, 61.3%	106 *BRCA*m (100%)	79 PlatS (74.5%)	600 mg bid	53.8% (43.8–63.5) PFI > 12 m: 73.9% (51.6–89.8) PFI 6–12 m: 62.5% (48.5–75.1) PFI < 6 m: 18.5% (6.3–38.1)
**VELIPAPRIB**							
Coleman et al. [138] (NCT01540565)	Phase 2	50	1: 28% 2: 36% 3: 36%	50 *BRCA*m (100%)	20 PlatS (40%)	400 mg bid	26% (16–38) PlatS: 35% (18–56) PlatR: 20% (9–36)
Steffensen et al. [132] (NCT01472783)	Phase 1–2	48	Median 4	48 *BRCA*m (100%)	13 Plat S (with PFI 6–12 m) Phase I: 4 (12.5%) Phase II: 9 (28.1%)	Phase I: escalation from 300 mg bid	Phase I: NR
						Phase II: 300 mg bid	Phase II:44% Plat S: 50% Plat R: 41%
**TALAZOPARIB**							
De Bono et al. [133] (NCT01286987)	Phase 1	34 Part 1: 23 Part 2: 11 *BRCA*m	Overall median: 2.5 Part 1 median: 4 Part 2: median 2	Part 1: NR Part 2: 11 (100%)	NR	Part 1: escalation 0.025–1.1 mg qd. Part 2: expansion. 1 mg qd.	Both cohorts: *BRCA*m: 48%. PlatS: 55% Plat R: 20%
							Part 2: 41.7%

**Table 2:** Randomized double-blind phase II and III trials assessing maintenance PARPi, showing PFS benefit, irrespective of *BRCA*m status, and non-significant or immature OS data. HRD: Homologous recombination deficiency. Non-HRD: Non homologous recombination deficient. HRDu: Homologous recombination deficiency unkown. LOHh: High loss of heterozygocity, LOHl: Low loss of heterozygocity; LOHu: Unkown loss of heterozygocity. NA: Not applicable. NR: Not reported.

	Trial Design	Patient Characteristics	*BRCA*/HRD	PFS	PFS (*BRCA*m)	PFS (*BRCA*wt/HRD)	PFS (*BRCA*wt/non-HRD)	OS
**OLAPARIB-FRONT LINE**								
Moore et al. [139] SOLO1 (NCT01844986)	Phase III. Olaparib vs. placebo.	N: 391 Serous 96% Front line.	g*BRCA*m 99%	At 3 years: 60% vs. 27% HR 0.3, *p* < 0.001	(Same as overall population)	NA	NA	Immature HR 0.95 (95% CI 0.6–1.53)
**OLAPARIB-PLATINUM SENSTIVE RECURRENCE**								
Ledermann et al. [140,141,142] STUDY19 (NCT00753545)	Phase II. Olaparib vs. placebo.	N: 265 High grade serous 91% ≤2 prior platinum 58%	g/s*BRCA*m 51%	8.4 vs. 4.8 months HR 0.35, *p* < 0.001.	11.2 vs. 4.3 months HR0.18, *p* < 0.0001	NA	7.4 vs. 5.5 months HR 0.54, *p* = 0.0075	29.8 vs. 27.8 months HR0.73, *p* = 0.025, NS.
Pujade-Lauraine et al. [143] SOLO2/ENGOT-OV21 (NCT01874353)	Phase III. Olaparib vs. placebo.	N: 295 Serous 91% ≤2 prior platinum 58%	g*BRCA*m 97%	19.1 vs. 5.5 months HR 0.3, *p* < 0.0001.	(Same as overall population)	NA	NA	Immature. HR: 0.8, *p* = 0.43
**NIRAPARIB-PLATINUM SENSTIVE RECURRENCE**								
Mirza et al. [144] ENGOT-OV16/NOVA (NCT01847274)	Phase III. Niraparib vs. placebo.	N: 553 Predominantly high grade serous. ≤2 prior chemotherapy 60%	g*BRCA*m: 203 (37%) g*BRCA*wt:350 (63%) HRD: 162 Non-HRD: 134 HRDu: 54	NR	21 vs. 5.5 months HR0.27, *p* < 0.001.	12.9 vs. 3.8 months HR0.38, *p* < 0.001	6.9 vs. 3.8 months HR 0.58. *p* = 0.02.	NR
**RUCAPARIB-PLATINUM SENSTIVE RECURRENCE**								
Coleman et al. [145] ARIEL 3 (NCT01968213)	Phase III. Rucaparib vs. placebo.	N: 564 Serous 95% ≤2 prior chemotherapy 63%	g/s*BRCA*m 196 (35%) LOHh 158 (28%) LOHl 161 (29%) LOHu 49 (9%)	10.8 vs. 5.4 months HR 0.36, *p* < 0.0001	16 vs. 5.4 months HR0.23, *p* < 0.0001	9.7 vs. 5.4 months HR0.44, *p* < 0.0001	6.7 vs. 5.4 months HR 0.58, *p* = 0.0049	NR

**Table 3:** Toxicity data from SOLO2, NOVA, ARIEL3 and FDA, EMA reports [143,144,145,146,147]. Cap: capsule, Tab: Tablet, GI: Gatrointestinal, ALT: Alanine aminotransferase, AST: aspartase aminotransferase, BID: twice daily, QD: once daily, Ch: chemotherapy.

	Olaparib	Niraparib	Rucaparib
Dosage	Cap: 400 mg bid Tab: 300 mg bid	300 mg qd	600 mg bid
FDA Approval	-2014: Single agent treatment. Cap formulation. g*BRCA*m, ≥3 prior Ch lines. -2017: Maintenance treatment. PlatS, and response to platinum Ch. Regardless of *BRCA*m status. -2018: First-line maintenance. Germline or somatic *BRCA*m carriers.	-2017: Maintenance treatment. PlatS relapse, and response to platinum Ch. Regardless of *BRCA*m status.	-2018: Maintenance treatment. PlatS, and response to platinum Ch. Regardless of *BRCA*m status.
EMA Approval	-2014: Maintenance treatment. Cap formulation. g/s*BRCA*m, PlatS relapse, and response to platinum. -2018: Maintenance treatment. Tablet formulation. PlatS relapse, and response to platinum Ch. Regardless of *BRCA*m status.	-2017: Maintenance treatment. PlatS relapse, and response to platinum Ch. Regardless *BRCA*m status.	-2018: Maintenance treatment. Germline or somatic BRCAm, PlatS and response to platinum Ch.
Toxicity			
Drug class toxicity	GI, fatigue, myelosuppression, headache, decrease appetite, creatinine increase, dyspnea.	GI, fatigue, myelosuppression, headache, decrease appetite, creatinine increase, dyspnea.	GI, fatigue, myelosuppression, headache, decrease appetite, creatinine increase, dyspnea.
≥10%, grade ≥3	Anemia.	Anemia, neutropenia, Thrombocytopenia.	Anemia, ↑ALT/AST.
Drug specific	-	Hypertension	↑ALT/AST
Other	Overall risk of MDS/AML 1%.		
